# Bone Mineral Density Changes in Long-Term Kidney Transplant Recipients: A Real-Life Cohort Study of Native Vitamin D Supplementation

**DOI:** 10.3390/nu14020323

**Published:** 2022-01-13

**Authors:** Yuri Battaglia, Antonio Bellasi, Alessandra Bortoluzzi, Francesco Tondolo, Pasquale Esposito, Michele Provenzano, Domenico Russo, Michele Andreucci, Giuseppe Cianciolo, Alda Storari

**Affiliations:** 1 Department of Medicine, University of Verona, 37129 Verona, Italy; 2 Nephrology and Dialysis Unit, Pederzoli Hospital, 37019 Verona, Italy; 3 Nephrology Unit, Ente Ospedaliero Cantonale, 6900 Lugano, Switzerland; antoniobellasi@gmail.com; 4 Rheumatology Unit, Department of Medical Sciences, University of Ferrara and St. Anna University-Hospital, 44124 Ferrara, Italy; alessandra.bortoluzzi@unife.it; 5 Nephrology, Dialysis and Renal Transplant Unit, IRCCS—Azienda Ospedaliero-Universitaria di Bologna, 40126 Bologna, Italy; francesco.tondolo@studio.unibo.it (F.T.); giuseppe.cianciolo@aosp.bo.it (G.C.); 6 Department of Internal Medicine, University of Genoa, 16132 Genoa, Italy; pasqualeesposito@hotmail.com; 7 Clinica Nefrologica, Dialisi, Trapianto, IRCCS Ospedale Policlinico San Martino, 16142 Genoa, Italy; 8 Nephrology and Dialysis Unit, Department of Health Sciences, “Magna Graecia” University, 88100 Catanzaro, Italy; michiprov@hotmail.it (M.P.); andreucci@unicz.it (M.A.); 9 Department of Public Health, University Federico II, 80100 Napoli, Italy; domenicorusso51@hotmail.com; 10 Nephrology and Dialysis Unit, St. Anna University-Hospital, 44124 Ferrara, Italy; a.storari@ospfe.it

**Keywords:** bone mineral disease, vitamin D, kidney transplantation, Z-score, T-score, femoral neck, lumbar vertebral bodies, DEXA, KDIGO guidelines

## Abstract

Vitamin D insufficiency has been associated with reduced bone mineral density (BMD) in kidney transplant patients (KTRs). However, the efficacy of vitamin D supplementation on BMD remains poorly defined, especially for long-term KTRs. We aimed to investigate the effect of native vitamin D supplementation on the BMD of KTRs during a 2-year follow-up. Demographic, clinical, and laboratory data were collected. BMD was evaluated with standard DEXA that was performed at baseline (before vitamin D supplementation) and at the end of study period. BMD was assessed at lumbar vertebral bodies (LV) and right femoral neck (FN) by a single operator. According to WHO criteria, results were expressed as the T-score (standard deviation (SD) relative to young healthy adults) and Z-score (SD relative to age-matched controls). Osteoporosis and osteopenia were defined as a T-score ≤ −2.5 SD and a T-score < −1 and a > −2.5 SD, respectively. Based on plasma levels, 25-OH-vitamin D (25-OH-D) was supplemented as recommended for the general population. Data from 100 KTRs were analyzed. The mean study period was 27.7 ± 3.4 months. At study inception, 25-OH-D insufficiency and deficiency were recorded in 65 and 35 patients. At the basal DEXA, the percentage of osteopenia and osteoporosis was 43.3% and 18.6% at LV and 54.1% and 12.2% at FN, respectively. At the end of the study, no differences in the Z-score and T-score gains were observed. During linear mixed model analysis, native vitamin D supplementation was found to have a negative nitration with Z-score changes at the right femoral neck in KTRs (*p* < 0.05). The mean dose of administered cholecalciferol was 13.396 ± 7.537 UI per week; increased 25-OH-D levels were found (*p* < 0.0001). Either low BMD or 25-OH-vitamin D concentration was observed in long-term KTRs. Prolonged supplementation with 25-OH-D did not modify BMD, Z-score, or T-score.

## 1. Introduction

With increased graft and patient survival, bone loss after kidney transplantation is becoming increasingly prevalent [1]. The expanding body of evidence suggests that bone loss portends poor survival [2]. Despite bone loss maybe being caused by immunosuppressive treatments [3] and a history of chronic kidney disease [4], vitamin D deficiency is regarded as a main causative factor, and therefore, supplementation is recommended. Indeed, the latest Kidney Disease: Improving Global Outcomes (KDIGO) guidelines on mineral metabolism management [5] recommend BMD testing to guide treatment with inactive vitamin D, calcitriol, and/or antiresorptive agents; in addition, depending on the 25-OH vitamin D (25-OH-D) plasma level, vitamin D supplementation is suggested as it is in the general population. However, the guidelines’ recommendations only refer to the first year after kidney transplant due to either a lack of data from longer observation periods or conflicting results of supplementation therapy [6,7,8,9,10].

Bone biopsy is the gold standard to accurately evaluate microarchitecture deterioration in the bone tissue in KTRs, but it is invasive, technically complex, not practical nor always available in clinical practice, and is often refused by patients [11]. An alternative, accurate, widespread, non-invasive, cost-effective method for assessing bone mineral density (BMD), a surrogate of bone mass, is a DEXA scan [12].

The present study aimed to investigate the impact of prolonged 25-OH vitamin D supplementation on BMD in a cohort of long-term KTRs, and, as secondary aims, to assess BMD and its potential association between BMD, demographic characteristics, and biochemical data.

## 2. Materials and Methods

This is a monocentric, observational, longitudinal study to assess the impact of 25-OH-D supplementation on BMD in KTRs. Patients were followed in the Nephrology Unit, Ferrara (Italy) University Hospital between 2013 to 2019 and met the following inclusion criteria: (1) being a recipient of a kidney from a cadaveric or living donor; (2) age ≥ 18 years; and (3) no therapy with inactive vitamin D sterols. Patients with parathyroidectomy and/or history of bone fractures and/or in treatment with bisphosphonate and/or calciomimetics were excluded. All patients gave their written informed consent, and the research protocol and the ethical approval was granted by the Hospital Ethics Committee for Human Research (Code: 356). All study procedures were conducted in accordance with the Declaration of Helsinki (Finland).

Patient data, including clinical characteristic and routine biochemistry, were collected from the digital patient archive.

Starting from the year 2013, 25-OH-D assay and BMD examination became part of scheduled KTR evaluation. An 25-OH-D assay was performed every three months to assess vitamin D levels to correct moderate (<30 ng/mL and ≥20 ng/mL) and severe insufficiency (<20 ng/mL and ≥10 ng/mL) or deficiency (<10 ng/mL) as recommended for general population [13,14]. Briefly, a course of 25,000 UI/week of cholecalciferol over 12 weeks followed by 1500 UI/day was administered.

Bone mineral density (BMD) was assessed via the Hologic Discovery DXA instrument (Hologic Inc., Waltham, MA, USA), which was available at the Rheumatology Unit of the same hospital. The reported precision (CV%) of the machine is ≤1%. All measurement were acquired using a standard phantom before each measurement. Bone mineral content (BMC) was calculated in grams (g), bone area was measured in centimetres squared (cm^2^), and BMD was measured in g/cm^2^ (BMC divided by the area).

According to the World Health Organization (WHO) criteria [15], results were expressed as the T-score (standard deviation [SD] relative to young healthy adults) and Z-score (SD relative to age-matched controls). The values were also expressed in percentile by comparing both the PR (peak reference) and AM (age-matched) groups. Normal bone density, osteopenia, and osteoporosis were defined as a T-score ≥ −1.0, T-score < −1 and >−2.5 SD, and T-score ≤ −2.5 SD at least one site, respectively.

According to the DEXA results, therapeutic recommendations were adopted at the discretion of the physician and in accordance with KDIGO clinical practice guidelines for the diagnosis, evaluation, prevention, and treatment of Chronic Kidney Disease-Mineral and Bone Disorder (CKD-MBD), which were issued in 2009 [14]. After a mean period of 2 years, BMD evaluation was repeated. The femoral neck (FN) and lumbar vertebral spine L1–L4 (LV) were measured to assess changes in bone mineral parameters.

Kidney function was evaluated with the estimated glomerular filtration rate (e-GFR) according to the equation from the Modification of Diet in Renal Disease Study [16]. BMI was calculated as weight (kg)/height^2^ (m^2^).

### Statistical Analysis

Continuous variables were expressed as the mean and standard deviation (SD) or median and interquartile range (IQR) based on their distribution and on categorical variables such as frequency (percentage). Based on the distribution of the continuous variables, the parametric (t-test) test was used to assess the within- as well as between-group differences. ANOVA was used to compare the laboratory and clinical differences among KTRs with normal BMD, osteopenia, or osteoporosis at LV and FN. Linear mixed model analysis [17] was implemented to test the impact of 25-OH-D use on Z-score, T-score, and BMD changes (dependent variables) and was adjusted for sex, age, presence of diabetes, steroid treatment, BMI [18], and time on dialysis. Z-score, T-score, and BMD changes were defined as Z-score, T-score, and BMD at follow-up and Z-score, T-score, and BMD at study inception. The same procedure was repeated for the femur and spine sites. Based on the limited data available, no sample size was calculated. Analysis was carried out using SPSS software (version 28, IBM Corp., Armonk, NY, USA), and statistical significance was considered as *p* < 0.05.

## 3. Results

### 3.1. Study Population

Data from 124 consecutive outpatients were collected. A total of 24 patients were excluded due to history of fractures (*N* = 8) and/or history of parathyroidectomy (*N* = 6), and/or therapy with calciomimetics (*N* = 11) and/or bisphosphonate (*N* = 13). The recruited patients were middle-aged (mean age: 54.4, SD: 11.8, males (69%) and females without a history of smoking (89%) and who predominately received haemodialysis treatment before kidney transplant (71%) (Table 1). The median transplant vintage was 73 (IQR 25.2–159) months (Table 1). Females were in their post-menopause stage (79%). The primary causes of kidney disease were mostly glomerulonephritis (43%) and Autosomal Dominant Polycystic Kidney Disease (20%). More than half of the KTRs (88/100) were on triple antirejection agents, including steroids (84%), calcineurin inhibitors (91%), mycophenolate mofetil (62%), azathioprine (11%), and mTOR inhibitors (9%).

At study inception, severe to moderate insufficiency and deficiency of 25-OH-D was identified in 65 and 35 patients, respectively. None of the participants had sufficient levels of 25-OH-D (Table 1). Calcitriol was used in 29 subjects.

### 3.2. First DEXA Assessment

Overall, the prevalence of osteopenia and osteoporosis was 43.3% (T-Score −1.65 with SD 0.33 and Z-score −1.10 with SD 1.12) and 18.6% (T-Score −3.05 with SD 0.50 and Z-score −2.20 with SD 0.58) at LV and 54.1% (T-Score −1.43 with 0.46; Z-score −0.84 with SD 1.48) and 12.2% (T-Score −2.34 with SD 1.29; Z-score −1.40 with SD 1.18) at FN, respectively (Table 2). DEXA data from LV (in three patients) and FN (in two patients) were not considered in the analysis due to poor quality [19].

Of interest, transplant vintage, gender, dialysis vintage, immunosuppressive therapy (steroids, calcineurin inhibitors, mycophenolate mofetil, azathioprine), eGFR, serum calcium levels, serum phosphate levels, and both 25-OH-D and iPTH concentrations were not different regardless of what the BMD was (normal, osteopenia, or osteoporosis at both measurement sites). In contrast, the osteoporotic lumbar spine and osteoporotic neck femur were found in patients with a lower BMI (23.39; SD 2.95) (F 3.17; df 2,94; *p* < 0.05) and who were of older age (58.33 SD 8.13) (F 4.10; df 2,95; *p* < 0.05) (Table 3), while a higher prevalence of osteoporosis at FN was present in patients in ongoing therapy with everolimus (*X*^2^ 2; N = 98; *p* = 0.006).

### 3.3. Follow-Up DEXA Assessment

After a mean of 27.7 (SD 3.4) months, a second DEXA examination was performed at both measurement sites for all patients. Among the osteoporotic KTRs, the mean femoral neck and vertebral spines T-scores were −2.97 (SD 0.23) and −3.08 (SD 0.36), respectively. Among the osteopenic KTRs, the mean femoral neck and vertebral spines T-scores were −1.61 (SD 0.67) and −1.82 (SD 0.45), respectively. Only 30 and 43 KTRs had normal BMD at the mean femoral neck and vertebral spines, respectively. DEXA data from LV (in four patients) and FN (in three patients) were not included in the analysis because of poor quality (Table 2).

No statistically significant differences in the Z-score, T-score, and BMD variations at the two measurement sites were observed in the entire sample (Table 4) or in the subgroups that had been divided according to WHO criteria (Supplementary Table S1).

However, during linear mixed model analysis, the positive interaction of inactive vitamin D supplementation with Z-score changes at the lumbar vertebral bodies did not reach statistical significance (*p* = 0.056) (Table 5 and Table 6).

In addition, no significant 25-OH-D effect was observed on the T-score and BMD changes at the two DEXA sites (Supplementary Tables S2–Table S5).

Supplementation therapy with cholecalciferol at a mean dose of 13.863 (SD 7.796) IU a week was administrated. Only one patient started therapy with calcitriol, and none of the patients started therapy with bisphosphonate. The 25-OH-D and calcium values increased to 28.8 (SD 9.0) ng/mL (t −11.45; df 190.73; *p* < 0.0001) and to 9.5 (SD 0.51) mg/dL (t −2.86; df 196; *p* < 0.005), respectively. No statistical significance changes in terms of the biochemical value (PTH and phosphorus) and eGFR were found. A sufficient level of 25-OH-D was found in almost half of the patients (45%), while a severe insufficiency or deficiency was only observed in 16 patients.

## 4. Discussion

In this study, we showed that inactive vitamin D supplementation was not associated with changes in bone mineral density over a median follow-up period of 2 to 3 years in a real-life cohort of long-term KTRs not treated with bisphosphonate and/or calcio-mimetics and who had never been supplemented with 25-OH vitamin D. A high percentage (80.6%) of patients fulfilled the criteria for low bone density at the first DEXA examination, with one-third reporting osteoporosis. Notably, the most common diagnosis was osteopenia both at the right femoral neck (54.1%) and at the lumbar vertebral bodies (43.3%). These results are consistent with previous studies [20,21,22] in which the prevalence of low BMD ranged from 49% to 80% in KTR cohorts depending on type of KT donor (living or deceased), the median time since KT (from 8 to 130 months), the length of follow-up (up to 2 years), and other demographic characteristics. Similarly, in a recent study by Jorgensen and coworkers, osteoporosis was detected in 46% (with the highest prevalence at the distal skeleton) of 141 unselected KTR who underwent a bone biopsy one year after transplantation, and this finding was independent of the type of bone turn-over abnormality [23]. Compared to other studies, our cohort was uniquely characterized by both a long transplant vintage (median: 70.5, IQR: 24.2–160.7 months) and a prolonged time interval between DEXA scans (mean: 27.7, SD: 3.4 months).

Regarding the vitamin status of our KTRs with the long-term duration of the graft, we found a high prevalence of 25-OH-D insufficiency (65%) and deficiency (35%) at baseline. The spontaneous tendency of 25-OH-D to increase over time was not observed and, as reported elsewhere [24], low levels of 25-OH-D remained stable independently of kidney graft age. The finding seems ubiquitous, and we could not identify any specific risk factors for decreased vitamin D serum levels. These results agree with a Danish study on 173 adult KTRs with a median graft vintage of 7.4 years (IQR: 3.3–12.7 years). As many as 80% of the patients had low serum levels of 25-OH-D, of whom 51% and 29% had vitamin D insufficiency and deficiency, respectively. However, our findings are in contrast with other reports, in which 25-OH-D insufficiency and deficiency were particularly prevalent during the first year after kidney transplant [25,26] and in selected populations, such as in the Africa-American population [27]. Of interest, another study of prevalent KTRs, which had a median post-transplant time of 6 years and included patients who had never been supplemented with vitamin D sterols presented 25-OH-D levels that were significantly higher when compared to the levels found in KTRs with a median post-transplant time of less than one year [28]. Although we cannot exclude a relationship between time from transplant and 25-OH-D levels, no differences in terms of vitamin D metabolites in patients with a longer graft duration was documented in a previous study [29]. These conflicting data could be partially explained by the fact that a successful kidney transplantation does not necessarily completely restore the ability to effectively metabolize vitamin D due to many factors, such as partial kidney function recovery, reduced sunlight exposure, inadequate dietary vitamin D intake, and/or the use of high corticosteroid dosage [30,31,32].

Surprising results emerged when analyzing the effect of vitamin D on BMD at the mixed model analysis after adjusting for confounding variables. Indeed, no relationship between 25-OH vitamin D replenishment and BMD was found. Although limited by the short duration of the follow-up period (up to 12 months) and by the renal transplant (up to 36 months), other studies have documented a slight to moderate increase in BMD values due to vitamin D supplementation in KTRs with decreased BMD [33,34].

However, our findings are consistent with those of an observational study with a long (3 years) follow-up, although that study was conducted in patients recruited 3 months after kidney transplantation. While lumbar spine BMD was decreased by 4% in patients taking alfacalcido, their Z-score and T-score values did not show significant changes with alfacalcidol treatment [35]. Additionally, in a cohort of 16 patients with a long transplant vintage (median: 118, SD:59 months), Cueto-Manzano et al. showed that the femoral neck and lumbar spine BMD changes of the treated group were not significantly different from controls. However, reduced bone formation was observed during bone biopsy after one year of therapy with 500 mg/d of calcium carbonate and 0.25 μg/d of 1,25-dihydroxyvitamin D3 [36].

Although it is difficult to draw correct conclusions on the effects of 25-OH vitamin D on BMD because of the different clinical characteristics of study cohorts, future prospective and interventional studies are required to assess the advantage of prolonged inactive vitamin D supplementation in long-term kidney transplant cohorts.

In our study cohort, no bisphosphonate therapy was started since bone biopsy had never performed to confirm the DEXA diagnosis of osteoporosis, as suggested by KDIGO 2009 [14]. Consequently, treatment decisions were mainly based on 25-OH-D levels. We could not detect an effect of these drugs on bones. However, a non-significant change in the prevalence of osteoporosis (33.6%) and osteopenia (46.7%) was found at the second DEXA measurement (*p* = 0.2). Our findings are consistent to those reported in a French prospective study that evaluated the 2-year changes in BMD in a cohort of KTRs recruited nine months post transplantation. Compared to baseline BMD status ( normal, osteopenia osteoporosis), no BMD change in no-bisphosphonate treatment KTRs (*p* = 0.1) was found [37]. Although the KDIGO clinical practice guidelines for the diagnosis, evaluation, prevention, and treatment of CKD-MBD issued in 2017 suggest the use of bisphosphonate within the first 12 months of transplantation, the safety and efficacy of bisphosphonate treatment on BMD in long-term patients should be evaluated by further prospective studies.

Regarding the effect of immunosuppressive therapy on bone mineral density, it is worth noting that treatment with everolimus was associated with a higher percentage of osteoporotic neck femur during the DEXA exam (*p* = 0.006). Although preclinical studies showed a protective effect of everolimus on bone health [38,39], our data are consistent with a retrospective study in which patients undergoing mTORs therapy had a higher risk of developing osteoporosis [40].

This study has strengths and limitations. Our results provide, for the first time, evidence of 25-OH-D vitamin supplementation having no effect on BMD, Z-score, and T-score at the femoral and lumbar sites in long term KTRs at 2 to 3 years follow-up. 

Some limitations of our study should be mentioned. Firstly, this is not a randomized study, which is the best study design to test the treatment efficacy, and the lack of control group does not allow us to generalize our results. Multicenter prospective studies on a larger KTR study cohort should be considered to provide more robust results since up until now, few studies on vitamin D on BMD have been conducted in long-term KTRs. Another limitation is represented by the fact that when our study started, KDIGO 2017 had not yet been published. However, by following the more restrictive use of bisphosphonate in accordance with KDIGO 2009, original evidence on BMD trends in untreated osteoporotic KTRs was provided. In addition, no data on FGF-23, a relevant but still debated marker of mineral metabolism for routine clinical practice [41], were available. Finally, we did not consider the lifestyles of the patients or the presence of skeletal fractures, nor did we assess additional risk factors for osteoporosis development and bone fractures, such as a family history of fractures or additional comorbidities that could affect bone loss [42,43,44].

## 5. Conclusions

Our study demonstrates that vitamin D supplementation was able to replenish 25-OH-D deficiency and insufficiency (84%) but did not affect the BMD, T-score, and Z-score over 2 years of follow-up in long-term kidney transplant recipients with a low BMD (80%) and 25-OH-vitamin (100%) status. Prospective and multicenter trials are required to shed light on the effects of vitamin D on BMD in KTRs after one year of transplant and to elucidate the role of vitamin D to correct mineral bone disorder in KTRs.

## Figures and Tables

**Table 1:** Demographic and biochemical data of kidney transplant recipients (KTRs) at baselines.

	KTRs (*n* = 100)	
Age, years *	53.29	(11.58)
Male, %	69.0	
Smoke, %	11.0	
Diabetes, %	10.0	
Race-Caucasian, %	94.0	
Weight, kg *	73.21	(12.22)
Height, cm *	170.88	(10.45)
BMI, kg/m^2^ *	24.71	(2.93)
HD Vintage Pre-KT, months *	28.33	(30.69)
KT pre-emptive, %	8.0	
KT vintage, months **	73.0	(25.2–159.0)
Systolic BP, mmHg *	130.25	(14.50)
Diastolic BP, mmHg *	78.10	(8.03)
HR, bpm *	72.98	(11.24)
Creatinine serum, mg/dL*	1.42	(0.53)
eGFR, ml/min/1.73 m^2^ *	53.22	(17.00)
Calcium serum, mg/dL *	9.37	(0.45)
Phosphorus serum, mg/dL *	3.20	(0.65)
25-OH Vitamin D, ng/mL *	14.85	(8.04)
iPTH, pg/mL **	83	(66.0–116.0)
Total Protein, g/dL *	6.61	(0.51)
Albumin, % *	58.70	(4.13)
LDH, U/L *	311.16	(66.20)
Urinary Calcium, mg/24 h *	112.06	(100.46)
Urinary Phosphorus, g/24 h *	0.69	(0.27)
Urinary Creatinine, g/24 h *	1.24	(0.45)
25-OH Vitamin D status:		
moderate insufficiency, %	26.0	
severe insufficiency, %	39.0	
deficiency, %	35.0	

* Data expressed as mean (standard deviation); ** median (inter quartile range); BP: blood pressure; BMI: body mass index; FC: HD: hemodialysis; HR: heart rate; GFR: estimated glomerular filtration rate; iPTH: intact parathormone; KT: kidney transplant; LDH: lactate dehydrogenase.

**Table 2:** DEXA data at time of first and second assessments for study population.

WHO Criteria	FN	Mean	SD	LV	Mean	SD
Normal BMD	T-score 1st	−0.473	0.709	T-score 1st	0.058	0.866
	T-score 2nd	−0.460	0.611	T-score 2nd	0.050	0.982
	*p*-value *	0.49		*p*-value *	0.96	
	Z-score 1st	0.261	0.790	Z-score 1st	0.671	1.038
	Z-score 2nd	0.393	0.763	Z-score 2nd	0.740	1.229
	*p*-value *	0.50		*p*-value *	0.79	
Osteopenia	T-score 1st	−1.700	0.450	T-score 1st	−1.652	0.338
	T-score 2nd	−1.611	0.680	T-score 2nd	−1.826	0.452
	*p*-value *	0.21		*p*-value *	0.06	
	Z-score 1st	−0.804	0.538	Z-score 1st	−1.102	1.125
	Z-score 2nd	−0.800	0.489	Z-score 2nd	−1.003	0.687
	*p*-value *	0.48		*p*-value *	0.63	
Osteoporosis	T-score 1st	−2.600	0.229	T-score 1st	−3.050	0.407
	T-score 2nd	−2.942	0.262	T-score 2nd	−3.085	0.365
	*p*-value *	0.002		*p*-value *	0.79	
	Z-score 1st	−1.575	0.430	Z-score 1st	−2.200	0.580
	Z-score 2nd	−1.786	0.433	Z-score 2nd	−2.236	0.693
	*p*-value *	0.227		*p*-value *	0.87	

* Unpaired T-test; BMD: bone mineral density; FN: right femoral neck; LV: lumbar vertebral bodies; SD: standard deviation; WHO: World Health Organization

**Table 3:** Relationship of demographic and biochemical data with bone mineral density status at lumbar vertebral bodies (LV) and right femoral neck (FN).

	WHO Criteria at LV			WHO Criteria at FN		
Age, years*	Normal (*n* = 37)	51.54	(11.04)	Normal (*n* = 33)	48.88	(10.64)
	Osteopenia (*n* = 42)	53.26	(12.95)	Osteopenia (*n* = 53)	54.42	(11.85)
	Osteoporosis (*n* = 18)	56.67	(9.28)	Osteoporosis (*n* = 12)	59.73	(6.87)
	Statistics °	*p* = 0.314		Statistics °	*p* = 0.01 °*	
Male, *n* (%)	Normal (*n* = 37)	27	(72.9)	Normal (*n* = 33)	23	(69.7)
	Osteopenia (*n* = 42)	28	(66.6)	Osteopenia (*n* = 53)	37	(69.8)
	Osteoporosis (*n* = 18)	12	(66.6)	Osteoporosis (*n* = 12)	7	(58.3)
	Statistics °°	*p* = 0.80		Statistics °°	*p* = 0.91	
Diabetes, *n* (%)	Normal (*n* = 37)	5	(13.5)	Normal (*n* = 33)	4	(12.1)
	Osteopenia (*n* = 42)	3	(7.1)	Osteopenia (*n* = 53)	5	(9.4)
	Osteoporosis (*n* = 18)	2	(11.1)	Osteoporosis (*n* = 12)	1	(8.3)
	Statistics °°	*p* = 0.64		Statistics °°	*p* = 0.91	
BMI, kg/m^2^ *	Normal (*n* = 37)	25.43	(2.89)	Normal (*n* = 33)	25.27	(3.20)
	Osteopenia (*n* = 42)	24.62	(2.74)	Osteopenia (*n* = 53)	24.72	(2.78)
	Osteoporosis (*n* = 18)	23.39	(2.95)	Osteoporosis (*n* = 12)	23.55	(2.62)
	Statistics °	*p* = 0.05 °*		Statistics °	*p* = 0.23	
HD Vintage Pre-KT, months *	Normal (*n* = 37)	24.97	(20.44)	Normal (*n* = 33)	25.00	(34.96)
	Osteopenia (*n* = 42)	23.95	(15.92)	Osteopenia (*n* = 53)	28.68	(28.96)
	Osteoporosis (*n* = 18)	39.44	(51.24)	Osteoporosis (*n* = 12)	40.00	(25.85)
	Statistics °	*p* = 0.11		Statistics °	*p* = 0.38	
KT vintage, months **	Normal (*n* = 37)	106.97	(109.95)	Normal (*n* = 33)	98.97	(93.45)
	Osteopenia (*n* = 42)	97.83	(85.74)	Osteopenia (*n* = 53)	112.28	(97.96)
	Osteoporosis (*n* = 18)	107.17	(102.11)	Osteoporosis (*n* = 12)	71.82	(85.97)
	Statistics °	*p* = 0.90		Statistics °	*p* = 0.42	
eGFR, ml/min/1.73 m^2^ *	Normal (*n* = 37)	51.76	(13.81)	Normal (*n* = 33)	53.30	(14.81)
	Osteopenia (*n* = 42)	55.98	(18.89)	Osteopenia (*n* = 53)	53.32	(17.73)
	Osteoporosis (*n* = 18)	49.50	(19.02)	Osteoporosis (*n* = 12)	52.36	(21.59)
	Statistics °	*p* = 0.33		Statistics °	*p* = 0.98	
Calcium serum, mg/dL *	Normal (*n* = 37)	9.37	(0.44)	Normal (*n* = 33)	9.35	(0.42)
	Osteopenia (*n* = 42)	9.43	(0.42)	Osteopenia (*n* = 53)	9.35	(0.48)
	Osteoporosis (*n* = 18)	9.32	(0.49)	Osteoporosis (*n* = 12)	9.45	(0.46)
	Statistics °	*p* = 0.63		Statistics °	*p* = 0.80	
Phosphorus serum, mg/dL *	Normal (*n* = 37)	3.11	(0.77)	Normal (*n* = 33)	3.11	(0.76)
	Osteopenia (*n* = 42)	3.19	(0.49)	Osteopenia (*n* = 53)	3.26	(0.63)
	Osteoporosis (*n* = 18)	3.30	(0.65)	Osteoporosis (*n* = 12)	3.10	(0.40)
	Statistics °	*p* = 0.60		Statistics °	*p* = 0.52	
25-OH Vitamin D, ng/mL *	Normal (*n* = 37)	13.24	(7.52)	Normal (*n* = 33)	12.84	(6.99)
	Osteopenia (*n* = 42)	14.73	(7.40)	Osteopenia (*n* = 53)	16.29	(8.60)
	Osteoporosis (*n* = 18)	17.70	(10.15)	Osteoporosis (*n* = 12)	13.72	(8.25)
	Statistics °	*p* = 0.16		Statistics °	*p* = 0.14	
iPTH, pg/mL **	Normal (*n* = 37)	119.00	(95.40)	Normal (*n* = 33)	106.87	(89.48)
	Osteopenia (*n* = 42)	87.77	(39.95)	Osteopenia (*n* = 53)	96.51	(57.20)
	Osteoporosis (*n* = 18)	102.38	(56.89)	Osteoporosis (*n* = 12)	112.0	(64.42)
	Statistics °	*p* = 0.14		Statistics °	*p* = 0.70	

* Data expressed as mean (standard deviation); ** median (inter quartile range); ° ANOVA test; °° Chi-Squared test; °* statistically significant; BMI: body mass index; eGFR: estimated glomerular filtration rate; HD: hemodialysis; iPTH: intact parathormone; KT: kidney transplant; WHO: World Health Organization.

**Table 4:** T-score, Z-score, and BMD (g/cm^2^) gains at 2 to 3 years follow-up in kidney transplant patients.

Gain T-Score				Gain Z-Score				Gain BMD			
Lumbar Spine	*p*	Femoral Neck	*p*	Lumbar Spine	*p*	Femoral Neck	*p*	Lumbar Spine	*p*	Femoral Neck	*p*
0.04 ± 0.49	0.64	−0.83 ± 0.33	0.63	0.98 ± 0.45	0.25	−0.32 ± 0.42	0.73	−0.02 ± 0.22	0.63	0.005 ± 0.06	0.89

**Table 5:** Mixed model effect of inactive vitamin D on Z-score in kidney transplant patients at right femoral neck.

Parameter	Estimate	Std. Error	df	t	Sig.	95% CI	
						LB	UB
Intercept	−4.206	1.215	160.804	−3.460	<0.001	−6.606	−1.805
Age	0.0191	0.011	127.615	1.670	0.097	−0.003	0.041
Sex	−0.571	0.268	144.941	−2.127	0.035 *	−1.101	−0.040
BMI	0.124	0.043	141.759	2.876	0.005 *	0.038	0.210
25-OH-Vitamin D	0.009	0.004	110.486	1.930	0.056	−0.001	0.019
Diabetes	−0.108	0.459	97.962	−0.236	0.814	−1.020	0.803
HD vintage	0.002	0.004	98.741	0.575	0.567	−0.006	0.011
Steroids	−0.135539	0.162	103.202	−0.837	0.405	−0.456	0.185

Dependent Variable: Z-score. * Statistically significant. BMI: body mass index; CI: confidence interval; HD: hemodialysis; LB: lower bound; UB: upper bound.

**Table 6:** Mixed model effect of inactive vitamin D on Z-score in kidney transplant patients at lumbar vertebral bodies.

Parameter	Estimate	Std. Error	df	t	Sig.	95% CI	
						LB	UB
Intercept	−2.462	0.743	149.884	−3.311	0.001	−3.931	−0.992
Age	−0.002	0.006	114.061	−0.415	0.679	−0.016	0.010
Sex	−0.311	0.160	123.858	−1.935	0.055 *	−0.629	0.007
BMI	0.094	0.025	121.308	3.684	<0.001 *	0.043	0.145
25-OH-Vitamin D	−0.005	0.003	122.614	−1.642	0.103	−0.013	0.001
Diabetes	0.362	0.259	98.616	1.400	0.165	−0.151	0.876
HD vintage	−0.003	0.002	98.390	−1.188	0.238	−0.008	0.002
Steroids	0.058	0.128	131.974	0.452	0.652	−0.196	0.312

Dependent Variable: Z-score. * Statistically significant. BMI: body mass index; CI: confidence interval; HD: hemodialysis; LB: lower bound; UB: upper bound.

## Data Availability

Not available.

## Supplementary Materials

The following supporting information can be downloaded at: https://www.mdpi.com/article/10.3390/nu14020323/s1, Table S1: T-score, Z-score, and BMD (g/cm^2^) gains at 2 to 3 years follow-up in kidney transplant patients according to WHO classification; Table S2: Mixed model effect of inactive vitamin D on T-score in kidney transplant patients at lumbar vertebral bodies; Table S3: Mixed model effect of inactive vitamin D on T-score in kidney transplant patients at right femoral neck; Table S4: Mixed model effect of inactive vitamin D on BMD (g/cm^2^) in kidney transplant patients at lumbar vertebral bodies; Table S5 Mixed model effect of inactive vitamin D on BMD (g/cm^2^) in kidney transplant patients at right femoral neck.
