# Supplementary material for: Bone Mineral Density Changes in Long-Term Kidney Transplant Recipients: A Real-Life Cohort Study of Native Vitamin D Supplementation
Source: Nutrients. 2022 Jan 13;14(2):323. doi: 10.3390/nu14020323 (PMC8780110; doi:10.3390/nu14020323)
Supplement: Supplementary file 1 [file nutrients-14-00323-s001.zip › nutrients-1537697-supplementary.pdf]

Bone Mineral Density Changes in Long-Term Kidney Transplant Recipients: A Real-Life Cohort Study of Native Vitamin D Supplementation

Supplementary Materials

Supplementary Table S1. T-score, Z-score, and BMD (g/cm^2) gains at 2 to 3 years follow-up in kidney transplant patients according to WHO classification.

	**Gain T-Score**				**Gain Z-Score**				**Gain BMD**			
WHO	Lumbar Spine	p	Femoral Neck	p	Lumbar Spine	p	Femoral Neck	p	Lumbar Spine	p	Femoral Neck	p
Normal	−0.04 (0.57)	0.96	−0.08 (0.38)	0.98	0.01 (0.47)	0.79	−0.12 (0.35)	0.51	−0.01 (0.08)	0.75	0.01 (0.05)	0.13
Osteopenia	0.07 (0.47)	0.56	−0.04 (0.28)	0.43	0.12 (0.45)	0.62	0.05 (0.45)	0.97	0.01 (0.07)	0.15	0.01 (0.07)	0.37
Ostoporosis	0.11 (0.35)	0.79	−0.21 (0.37)	0.002	0.18 (0.39)	0.87	−0.15 (0.40)	0.22	−0.10 (0.49)	0.31	−0.04 (0.06)	0.52

Supplementary Table S2. Mixed model effect of inactive vitamin D on T-score in kidney transplant patients at lumbar vertebral bodies.

**Parameter**	**Estimate**	**Std. Error**	**df**	**t**	**Sig.**	**95% CI**	
						**LB**	**UB**
Intercept	−3.009	1.018	184.909	−2.953	0.004	−5.019	−0.998
Age	−0.009	0.010	159.924	−0.939	0.349	−0.029	0.010
Sex	−0.103	0.229	180.321	−0.449	0.654	−0.555	0.349
BMI	0.099	0.037	179.684	2.686	0.008 *	0.026	0.173
25-OH-Vitamin D	0.003	0.003	110.294	1.183	0.239	−0.002	0.010
Diabetes	0.267	0.442	98.485	0.605	0.547	−0.609	1.144
HD vintage	0.003	0.004	97.805	0.863	0.390	−0.004	0.012
Steroids	−0.137	0.104	93.845	−1.311	0.193	−0.345	0.070

Dependent Variable: T-Score. * Statistically significant. BMI: body mass index; CI: confidence interval; HD: hemodialysis; LB: lower bound; UB: upper bound.

Supplementary Table S3. Mixed model effect of inactive vitamin D on T-score in kidney transplant patients at right femoral neck.

**Parameter**	**Estimate**	**Std. Error**	**df**	**t**	**Sig.**	**95% CI**	
						**LB**	**UB**
Intercept	−1.777	0.772	150.244	−2.302	0.023	−3.303	−0.252
Age	−0.034	0.007	114.766	−4.757	<0.001 *	−0.048	−0.019
Sex	−0.154	0.167	124.839	−0.924	0.357	−0.485	0.176
BMI	0.093	0.026	122.209	3.480	<0.001 *	0.040	0.146
25-OH-Vitamin D	−0.003	0.003	122.234	−0.885	0.378	−0.010	0.004
Diabetes	0.307	0.270	98.815	1.137	0.258	−0.228	0.842
HD vintage	−0.002	0.002	98.555	−0.839	0.403	−0.007	0.003
Steroids	0.056	0.132	130.925	0.428	0.669	−0.204	0.317

Dependent Variable: T-Score. * Statistically significant. BMI: body mass index; CI: confidence interval; HD: hemodialysis; LB: lower bound; UB: upper bound.

Supplementary Table S4. Mixed model effect of inactive vitamin D on BMD (g/cm^2) in kidney transplant patients at lumbar vertebral bodies.

Parameter	Estimate	Std. Error	df	t	Sig.	95% CI	
						LB	UB
Intercept	0.784	0.180	143.738	4.333	<0.001	0.426	1.141
Age	−0.002	0.001	96.064	−1.548	0.125	−0.005	0.001
Sex	0.043	0.035	99.687	1.212	0.228	−0.027	0.114
BMI	0.010	0.005	101.151	1.852	0.067	−0.0007	0.022
25-OH-Vitamin D	−0.0005	0.001	151.795	−0.422	0.674	−0.002	0.001
Diabetes	0.011	0.056	93.098	0.204	0.839	−0.100	0.122
HD vintage	0.0004	0.0005	99.892	0.778	0.438	−0.0006	0.001
Steroids	−0.0005	0.039	164.620	−0.015	0.988	−0.079	0.078

Dependent Variable: BMD (g/cm^2). BMI: body mass index; CI: confidence interval; HD: hemodialysis; LB: lower bound; UB: upper bound.

Supplementary Table S5. Mixed model effect of inactive vitamin D on BMD (g/cm^2) in kidney transplant patients at right femoral neck.

Parameter	Estimate	Std. Error	df	t	Sig.	95% CI	
						LB	UB
Intercept	0.607	0.098	149.014	6.189	<0.001	0.413	0.801
Age	−0.004	0.0008	111.596	−4.590	<0.001 *	−0.005	−0.002
Sex	0.018	0.021	120.023	0.867	0.388	−0.023	0.059
BMI	0.011	0.003	117.902	3.397	<0.001 *	0.004	0.018
25-OH-Vitamin D	−0.0002	0.0005	125.972	−0.431	0.667	−0.001	0.0007
Diabetes	0.070	0.033	98.574	2.096	0.039 *	0.003	0.136
HD vintage	−0.0002	0.0003	98.523	−0.750	0.455	−0.0009	0.0004
Steroids	0.024	0.017	138.802	1.345	0.181	−0.011	0.059

Dependent Variable: BMD (g/cm^2). * Statistically significant. BMI: body mass index; CI: confidence interval; HD: hemodialysis; LB: lower bound; UB: upper bound.
